# Reprogramming of glucose metabolism in pancreatic cancer: mechanisms, implications, and therapeutic perspectives

**DOI:** 10.3389/fimmu.2025.1586959

**Published:** 2025-06-24

**Authors:** Yan Zhang, Wancheng Li, Jubao Niu, Zeyang Fan, Xin Li, Hui Zhang

**Affiliations:** ^1^ The Second Clinical Medical School, Lanzhou University, Lanzhou, China; ^2^ Department of General Surgery, The Second Hospital of Lanzhou University, The Second Clinical Medical School, Lanzhou University, Lanzhou, China

**Keywords:** pancreatic cancer, glucose metabolism, tumor microenvironment, treatment resistance, therapeutic strategy

## Abstract

As a typical pathological feature of pancreatic ductal adenocarcinoma, reprogramming of glucose metabolism synergistically drives the tumorigenesis and development process through molecular mechanisms such as regulating the expression of driver genes, modifying key functional proteins, triggering mitochondrial metabolism abnormality, and remodeling the tumor microenvironment. It is worth noting that this metabolic remodeling phenomenon is significantly associated with the formation of chemoresistance. Based on the latest research progress, this paper systematically describes the molecular basis of glucose metabolic reprogramming in pancreatic cancer, drug resistance characteristics and its targeted intervention strategies, and provides a theoretical framework for the research and development of innovative drugs.

## 1 Introduction

Pancreatic ductal adenocarcinoma (PDAC), the main subtype of pancreatic cancer (accounting for more than 90% of cases), is highly approachable in terms of morbidity and mortality, and is projected to jump to the second place in cancer-related deaths in 2030 (1). Due to the lack of early diagnostic markers, more than 90% of patients are diagnosed at an advanced or metastatic stage, with a 5-year survival rate of only 11% (2). Although surgery can prolong the survival of some patients, the rate of postoperative metastasis is extremely high, and existing treatments, such as chemotherapy, have limited effectiveness and are accompanied by serious side effects in advanced patients (3).

Malignant progression of PDAC is closely related to metabolic reprogramming. Tumor cells continuously acquire energy through aerobic glycolysis (Warburg effect (4)), preferring glycolysis over oxidative phosphorylation even under hypoxic conditions. This metabolic pattern not only provides adenosine triphosphate(ATP) and macromolecular precursors, but also acidifies the tumor microenvironment (TME) by releasing lactate, which in turn promotes immune escape, chemoresistance, and metastasis (5).

Therefore, a more comprehensive exploration of aerobic glycolysis will help to unravel the mechanisms of PDAC progression and point to potential therapeutic avenues. This review provides insights into the mechanisms of reprogramming of PDAC glycolysis, the tumor immune microenvironment, subtype specificity, therapeutic resistance, and targeted therapies.

## 2 Mechanisms of reprogramming glucose metabolism in pancreatic cancer

### 2.1 Metabolite-based metabolic reprogramming in pancreatic cancer

Metabolites are intermediates of cellular metabolism, and a variety of metabolites have been reported to be involved in the regulation of multiple signaling pathways in PDAC development. Cancer cells draw large amounts of energy from glycolysis and produce large amounts of lactate even under aerobic conditions (6). Lactate, an abundant cancer metabolite, is an end product of glycolysis. Enhanced glycolysis and accumulation of lactate is a common feature of many types of cancers. Lactate is the main carbon source of the tricarboxylic acid(TCA) cycle and therefore a major energy source, glucose transporters (GLUTs) and lactate dehydrogenase (LDHA) play an important role in the high-speed conversion of glucose to lactate play an important role in the conversion of glucose to lactate (7). In normal cells, most of the pyruvate enters the mitochondria for oxidation and participates in the generation of ATP after TCA, whereas in pancreatic cancer cells, there is an enhancement of glycolysis and a significant increase in the rate of production of pyruvate, which is converted to lactate. The production of lactate is accompanied by the production of NADH (nicotinamide adenine dinucleotide hydrogen), and pancreatic cancer cells gain energy through enhanced glycolysis to produce large amounts of NADH. Lactate not only provides energy for pancreatic cancer cells, but also suppresses immune cells by altering the tumor microenvironment. Lactate is exported from cancer cells via the monocarboxylate transporter (MCT), and the exported lactate is a potential substrate for the TCA cycle of the surrounding cells to meet their energy requirements. In response to MCT, the investigators provided a therapeutically relevant approach to reduce cGAS lactation by blocking MCT1 (8), thereby altering the pH of the tumor microenvironment (TME), and thus the TME of the cancer (9). Accumulation of cancer cell-derived lactate in the TME has been shown to impair cytotoxic T-lymphocyte cytokine production and proliferation by blocking and disrupting T-cell metabolism via lactate efflux and lactate from cancer cells stimulates production of IL-6 by cancer-associated fibroblasts (CAFs), synergistically impairing cytotoxic lymphocyte function (10).However, it has been found that lactate-pretreated *in vitro* CD8 + T cells effectively inhibit tumor growth when overtransferred to hormonal mice, and that lactate increases stemness and enhances anti-tumor immunity in CD8 + T cells. This suggests that the effects of lactate on tumors and immune cells are highly complex and difficult to interpret (11).

**Figure 1:**
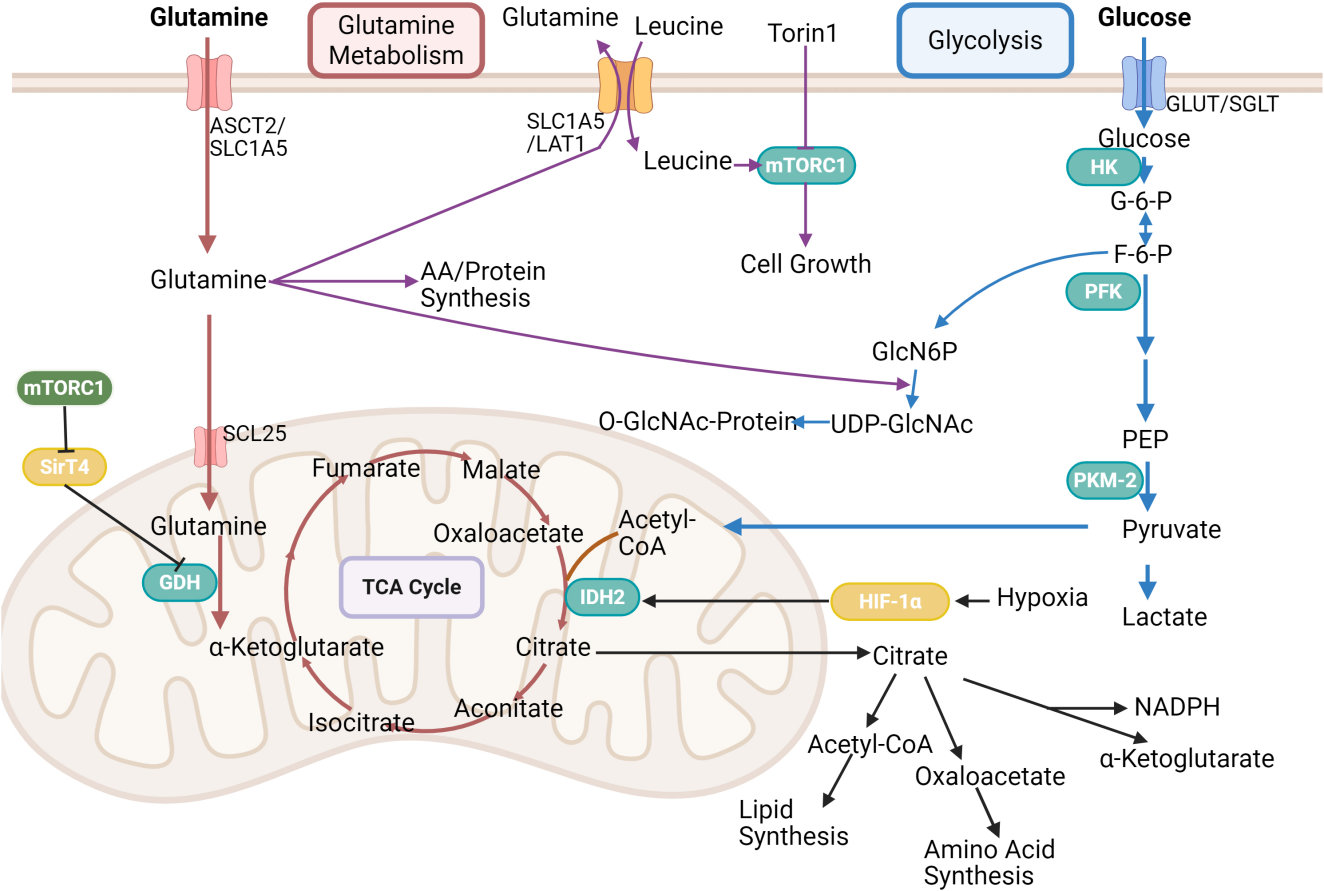
This figure presents the link between glycolysis, TCA cycle and glutamine metabolism. Glutamine is transported into cells via ASCT2/SLC1A5, and leucine activates mTORC1 for cell growth, which is inhibited by Torin1. Glutamine is partly used for amino acid/protein synthesis, partly into the mitochondria via GDH to participate in the tricarboxylic acid cycle, which is regulated by HIF-1α and Sirt4. In glycolysis, glucose undergoes a series of enzymatic reactions to form pyruvate, which can enter the tricarboxylic acid cycle, and its product, citric acid, is used in lipid and amino acid synthesis, etc. It can also participate in the hexosamine biosynthetic pathway. Created in https://BioRender.com.

It is important to note that in normal lung tissue and lung cancer, the largest TCA contribution comes from lactate, whereas in PDCA, glutamine contributes more (12). In PDCA, glutamine not only participates in the TCA cycle to generate energy, but also supports cancer cell proliferation by regulating the supply of carbon sources, and glutamine plays a more critical role as an important metabolic substrate. This central role relies on the optimization of glutamine catabolic pathways by mitochondrial metabolic reprogramming. The key initiating step in glutamine metabolism is catalyzed by glutaminase (GLS), which converts glutamine to Glu. Glu is in turn converted to α-ketoglutarate (α-KG), which enters the TCA cycle, either by glutamate dehydrogenase activity or by the action of transaminases (e.g., aspartate aminotransferase, glutamic oxaloacetic acid transaminase 1/2, i.e., GOT1/GOT2). PDAC cells are particularly preferred to metabolize glutamine via GOT1/GOT2-mediated transamination reactions, which help maintain the levels of nicotinamide adenine dinucleotide phosphate (NADPH) and nicotinamide adenine dinucleotide (NAD) pools, thereby supporting both tumor growth and intracellular redox homeostasis (13). The versatility of Glu goes far beyond energy metabolism. It is an indispensable substrate for the synthesis of lipids, nucleotides and proteins. More importantly, glutamine provides a key nitrogen source and/or carbon skeleton for the biosynthesis of a wide range of amino acids, including asparagine, glutamate, proline, aspartate, alanine, glycine, serine, cysteine, and ornithine (14). Among other things, it was found that there is a significant dependence of PDAC on the flow of glutamine metabolism to the ornithine synthesis pathway. This process is mediated by ornithine aminotransferase (OAT) and supports polyamine synthesis essential for tumor growth (15). Oncogenic genetic alterations driving the development of PDAC are central regulators of reprogramming of glutamine metabolism (16). Through its powerful transcriptional regulation, c-MYC directly binds to and up-regulates the gene expression of high-affinity glutamine transporter proteins, thereby significantly enhancing cellular uptake of glutamine (17). On the other hand, the tumor suppressor TP53 targets and regulates the expression of GLS2, which promotes mitochondrial respiration and ATP synthesis by increasing glutamate and α-KG production, and enhances cellular antioxidant defenses and protects against oxidative stress-induced apoptosis by up-regulating glutathione levels and decreasing reactive oxygen species (ROS) levels (18, 19). Dissecting the complex network of glutamine metabolism will provide a theoretical foundation for the development of therapeutic strategies that target metabolic vulnerabilities, while driving synergistic innovation of metabolic interventions with existing therapies such as immunotherapy. The interactions among glycolysis, the TCA cycle, and glutaminolysis are schematically summarized in **Figure 1**.

Tissues obtain ATP from two pathways - glycolysis and the TCA cycle coupled to the electron transport chain. Most energy in mammals is produced through TCA metabolism. Glycolytic flux is increased in tumors compared to healthy tissue, but this increase is not sufficient to compensate for the low TCA flux for ATP production. As a result, solid tumors typically produce ATP at a slower-than-normal rate, rather than the high metabolism that is often assumed. In mouse pancreatic cancer, this can be achieved by down-regulation of protein synthesis, which is one of the major energy expenditures of the tissue. In solid tumors, cancer cells lose some tissue-specific function and grow uncontrollably despite their limited ability to produce ATP (20). NADH plays an important role in the TCA cycle, acting as a donor to the electron transport chain and supporting ATP production. Although the flux of the TCA cycle is low in pancreatic cancer cells, it is still involved in cellular energy production. Since cancer cells rely primarily on glycolysis for energy production, NADH maintains the NAD+/NADH ratio through the action of lactate dehydrogenase and other regulatory enzymes. The NAD+/NADH balance directly affects cellular metabolic pathways, especially in the presence of enhanced glycolysis and decreased TCA cycle function.

### 2.2 Metabolic reprogramming in pancreatic cancer based on gene expression regulation

#### 2.2.1 Driver genes

A feature of PDAC is the prevalence of oncogenic mutations in the Kras gene, which plays a key role in the development and maintenance of pancreatic tumors and is involved in cell proliferation and survival. The Kras mutation was identified at an early stage of PDAC development (PanIN1) (21), suggesting that it is important for tumorigenesis and that tumor progression requires other genes for additional mutations. Oncogenic Kras is associated with regulation of the TME and pro-tumorigenic cell recruitment, leading to tumor invasion and metastasis (22, 23). The important role of Kras in the maintenance of an immunosuppressive TME and the opportunity to combine Kras targeting with immunotherapy to achieve a sustained response to therapy. It has been shown that Kras (G12D) plays an important role in controlling pancreatic cancer metabolism by stimulating glucose uptake and directing glucose intermediates into hexosamine biosynthesis and the pentose phosphate pathway (PPP). These studies have also revealed that oncogenic Kras promotes ribose biogenesis (24). Newly approved and emerging Kras G12C inhibitors can only benefit a small percentage of pancreatic cancer patients, and there are no approved drugs targeting the major PDAC mutations Kras G12D and G12V (25).

Robert A. Weinberg emphasized the important role of genomic instability in the characterization of cancer and recognized metabolic reprogramming as a novel feature associated with activated oncogenes (26). Although aerobic glycolysis clearly affects cancer cell survival, it is controlled by proteins involved in cellular programs that are central to other hallmarks of cancer. The Warburg effect may constitute a certain phenotype driven by genomic alterations. Epigenetic dysregulation leads to aberrant gene expression, which promotes malignant transformation and progression of tumors in several ways. SET structural domain 2 (SETD2) is a single histone methyltransferase responsible for catalyzing the trimethylation of histone H3 lysine 36 (H3K36me3), which is somatically mutated in a variety of malignant tumors, including PDAC (27). Hotspot mutations in SF3B1 (splicing factor 3b subunit 1) have been observed in a variety of cancers and these mutations result in a large number of aberrant mRNA splices, which are strongly associated with tumorigenesis. Specifically, the SF3B1 K700E mutation is tightly associated with tumor growth in PDAC. The SF3B1 K700E mutation promotes glycolysis in tumor cells, increasing glucose consumption, lactate release, and extracellular acidification. The mutation activates the glycolysis regulator c-Myc through aberrant splicing of the protein phosphatase 2 regulatory subunit B’alpha(PPP2R5A) gene and through post-translational regulation. This finding provides a potential therapeutic strategy for targeting PDAC with the SF3B1 K700E mutation (28).

In addition, leptin, an adipokine, is significantly elevated in obese patients and plays an important role in several biological processes in tumors. A study demonstrated that *in vitro* leptin treatment significantly promoted cell proliferation, glucose uptake, and lactate production in human PDAC and healthy pancreatic cells in a dose-dependent manner and was accompanied by an increase in the expression of the glycolytic enzymes hexokinase 2 (HK2) and glucose transporter protein 1 (GLUT1) (29). TWIST1 is an important regulator of aerobic glycolysis in PDAC. And its genetic silencing significantly inhibited the glycolytic phenotype of PDAC cells, e.g., by decreasing glucose uptake, lactate production, and extracellular acidification rates. Notably, induction of TWIST1 expression promotes the Warburg effect in PDAC cells, i.e., enhanced aerobic glycolysis (30). In addition, p38γ connects Kras oncogene signaling with the Warburg effect via 6-phosphofructo-2-kinase/fructose-2,6-biphosphatase 3(PFKBF) and GLUT2, which in turn promotes PDAC (31). In general, Forkhead box Q1(FOXQ1) promotes LDHA transcription and aerobic glycolysis. High FOXQ1 expression appears to be associated with poor clinical outcomes and has been identified as an independent prognostic marker for poor survival in pancreatic, colorectal, lung, gastric, and hepatocellular cancers (32). There are some exceptions. Several studies have reported increased expression of FOXQ1 in breast cancer cells (33). However, in certain subtypes of breast cancer, i.e., HER2-positive breast cancer and ductal breast cancer, the level of FOXQ1 expression is lower compared to healthy tissue and triple-negative breast cancer (34). In these cancers, high FOXQ1 expression was found to be associated with a more favorable clinical outcome. FOXQ1 can also apparently act as a tumor suppressor in certain types of cancers, and its loss in these cancers results in a worse prognosis.

#### 2.2.2 Non-coding RNA

Non-coding RNAs are a class of RNAs that do not code for proteins but are involved in a variety of cellular functions, including the regulation of glycolysis. Non-coding RNAs, including microRNAs (miRNAs), long chain non-coding RNAs (lncRNAs) and circular RNAs (circRNAs), have different functions in tumor cells (35). It has been shown that miRNAs, lncRNAs and circRNAs are involved in the development of pancreatic cancer cells, in part by regulating glycolysis (36).

MiRNAs are a class of non-coding RNAs that are approximately 22 nucleotides in length. Studies have shown that several miRNAs are dysregulated in PDAC and can regulate glycolysis in PDAC and a variety of genes in tumor cells. Most of the miRNAs identified in recent studies were able to inhibit glycolysis in PDAC. MicroRNA323a (miR-323a) was downregulated in pancreatic cancer tissues and cells and was enriched in the glucose metabolism pathway. MiR-323a was downregulated within pancreatic cancers and acted as a tumor-suppressor miRNA by inhibiting cancer cell proliferation and glycolysis, and targeting HK-2 for its Tumor suppressor role (37). MiR-30d is a functional and clinical tumor suppressor gene in PDAC. MiR-30d was found to be a novel target of YT521-B homology (YTH) structural domain protein 1(YTHDC1). Through m6A modification. MiR-30d suppresses pancreatic tumorigenesis by inhibiting aerobic glycolysis (38).

Long non-coding RNAs (lncRNAs) regulate gene expression through a variety of mechanisms and play crucial roles in a wide range of cellular processes such as chromatin remodeling, embryonic development, cell differentiation, energy metabolism and tumorigenesis (39). Several dysregulated lncRNAs with oncogenic activity have been identified in PDAC, such as LINC00673, UNC5B-AS1, DICER1-AS1, LINC00261, and lncRNAmIR210hg. LINC01559 and UNC5B-AS1 significantly inhibited the glycolytic activity of BxPC-3 cells, with glucose utilization decreased, and the rates of lactate production and extracellular acidification were reduced. LINC01559 and UNC5B-AS1 expression was also closely correlated with the mRNA levels of many glycolytic components of the glycolytic pathway, suggesting a regulatory role of LINC01559 and UNC5B-AS1 in PDAC glycolysis (40, 41). LncRNA DICER1-AS1 expression is down-regulated in PDAC and negatively correlates with glycolytic gene expression. DICER1-AS1 promotes the transcription of its justice gene, DICER1, by recruiting the transcription factor YY1 to the DICER1 promoter. Meanwhile, DICER1 promoted the maturation of miR-5586-5p, which inhibited the expression of glycolytic genes including LDHA, HK2, PGK1 and SLC2A1. Notably, reciprocal feedback between the N6-methyladenosine reader YTHDF3 and the lncRNA DICER1-AS1 promotes glycolysis in pancreatic cancer by inhibiting the maturation of miR-5586-5p (42). LINC00261 is down-regulated in pancreatic cancer tissues and cell lines. The down-regulation of LINC00261 is driven by the promoter regions of the CpG islands hypermethylation and EZH2-mediated trimethylation of histone H3 lysine 27. In addition, LINC00261 exerts its biological function by binding to miR-222-3p to activate the HIPK2/ERK/c-Myc pathway. LINC00261 also reduces c-Myc expression by sequestering IGF2BP1 (43). LncRNA mIR210hg is abnormally up-regulated within pancreatic cancer and is a PDAC aggressive and a key oncogenic regulator of glycolysis. Knockdown of MIR210HG significantly suppressed the aggressive phenotype of pancreatic cancer cells and inhibited the growth of xenograft tumors. More importantly, MIR210HG knockdown inhibited pancreatic cancer cell glycolysis by regulating the expression of glycolysis-associated HK2 and pyruvate kinase muscle isoform M2 (PKM2). MIR210HG affects pancreatic cancer cell phenotypes including proliferation, invasion, migration, and glycolysis by regulating the miR-125b-5p/HK2/PKM2 axis (44). LncRNAs, in addition to having a role in PDAC development, also play an important role in GEM resistance, e.g., HIF1A-AS1 significantly up-regulates lncRNAs in GEM-R pancreatic cancer cells and triggers glycolysis-associated GEM resistance by regulating the translation of the justice gene HIF-1α (45).

Circular RNAs (circRNAs) are a group of predominantly non-coding RNAs produced by reverse splicing without 5’ and 3’ end structures (46, 47). Hsa_circRNA_103809 (circ_0072088) has emerged as an emerging tumor regulator in human cancers and was identified as one of the most aberrantly expressed circRNAs in PDAC patients with one of the most aberrantly expressed circRNAs. Circ_0072088 plays an oncogenic role in the malignant progression and glycolysis of PDAC cells through the circ_0072088/miR-545-3p/SLC7A11 pathway (48). CircPUM1 activates miR-200c-3p through phagocytosis of the PI3K/AKT signaling pathway and promotes PDAC progression (49). Circ_03955 functions as a tumor promoter through the miR-3662/HIF-1α axis, providing new perspectives on the treatment of PDAC (50). CircLIPH may exert its oncogenic biological effects by activating the miR769-3p/GOLM1/PI3K/AKT/mTOR axis, whereas si-circLIPH effectively inhibited the expression of circLIPH and suppressed tumor growth through apoptosis *in vivo (* 51). CircSLIT2 is significantly upregulated in PDAC tissues and cells, and circSLIT2/miR-510-5p/c-Myc/LDHA axis is involved in aerobic glycolysis and carcinogenesis in PDAC (52).

### 2.3 Key proteins and their modifications

Almost all enzymes in glycolysis as well as regulatory proteins play a dual functional role in the progression of PDAC. These enzymes or related proteins typically exert catalytic activity in the cytoplasm and regulate transcription factors in the nucleus. All are critical in the proliferation, invasion, migration and metastasis of PDAC, and some new strategies for the treatment and detection of PDAC can be developed from this information. LDHA catalyzes the conversion of pyruvate to lactate in the final step of glycolysis, thus becoming a key regulator of the “Warburg effect”. LDHA is modified or overexpressed in PDAC tissues, and LDHA is post-translationally palmitoylated at cysteine 163 by ZdhHC9, which promotes the enzyme activity and lactate production, and reduces reactivity. lactate production and reduced ROS production. ZdhHC9 expression is upregulated in pancreatic cancer and correlates with poor patient prognosis (53). FOXQ1 promotes LDHA transcription and facilitates aerobic glycolysis (54). Modulation of LDHA expression or activity affects PDAC cell migration and globule growth, decreases metalloproteinases and cancer stem cell-like cell marker (i.e., CD133+) expression (55).LDHA overexpression decreased phosphorylation of the metabolic regulator AMPK and promoted downstream mTOR phosphorylation in PDAC cells, enhancing PDAC cell proliferation, tumor stem cell proliferation, invasion, and metastasis (56). When tumor growth exceeds its oxygen supply, upregulation of LDHA ensures ATP synthesis, increases resistance to oxidative stress by promoting DNA repair and NADPH production, and reduces ROS by inhibiting mitochondria, favoring Epithelial-Mesenchymal Transition(EMT) epithelial-mesenchymal transition and angiogenesis by starving neighboring cells for nutrients and evading the immune system to create space (57). Knockdown of LDHA expression prevents the growth of PANC-1 and CFPAC-1 cell growth (58). Expression of ubiquitin carboxy-terminal hydrolase L3 (UCHL3) was significantly increased in pancreatic cancer tissues and cells, and knockdown of UCHL3 significantly inhibited cell viability and aerobic glycolysis. UCHL3 promotes LDHA expression and can be reduced by shFOXM1, and low-expression of LDHA partially reversed the inhibition of aerobic glycolysis induced by overexpression of UCHL3, UCHL3 may be a potential diagnostic and therapeutic target for the treatment of cancer (59). LDHA inhibition attenuates tumor cell proliferation and promotes proliferation and infiltration of anti-tumor T lymphocytes by reducing lactate production. However, targeting LDHA was mildly effective in tumor tissues lacking CD8+ T lymphocytes. In addition, LDHA has been found to have other roles in cellular metabolism, such as catalyzing the production of 2HG, αHB, and metabolites that epigenetically influence disease progression (60). However, it was found that LDHB expression was not associated with the prognosis of PDAC (61).

The regulatory role of glycolysis-related proteins, such as fructose-1,6-bisphosphatase (FBP1), which encodes a rate-limiting gluconeogenic enzyme, has been reported to play a role in tumor suppression in many cancers (62). Deficiency in FBP1 expression has been associated with a poor prognosis in patients with pancreatic and hepatocellular carcinomas (63, 64). In particular, a previous study demonstrated that inhibition of FBP1 in PDAC leads to tumor progression by altering glucose metabolism (63). GLUT1 plays a critical role in PDAC progression, and Forkhead Box D1(FOXD1)enhances GLUT1 expression by regulating aerobic glycolysis, which promotes proliferation, invasion, and metastasis of PDAC cells (65). UBR5 is an E3 ubiquitin ligase that has been implicated in the regulation of metabolism, proliferation, and apoptosis (66). UBR5-induced aerobic glycolysis is dependent on FBP1 in pancreatic cancer cells and there is a significant negative correlation between the levels of UBR5 and FBP1. UBR5 regulates FBP1 expression through modulation of C/EBPα, directly binding to C/EBPα, and facilitating its ubiquitylation and degradation of FBP1 expression (67).

Histones are involved in the regulation of a variety of physiological functions, and post-translational modifications of histones (e.g., lactylation, acetylation, succinylation, etc.) play an important role in the development of diseases. Metabolic reprogramming and epigenetic remodeling are closely related and regulate each other. For example, microglia exhibit significant enrichment in brain specimens from Alzheimer’s disease patients for histone H4 lysine 12 lactoylation, which targets the promoter regions of genes associated with glycolysis, thereby inducing their transcription and promoting lactate production. This establishes a positive feedback loop involving metabolism-epigenetics-metabolism, which exacerbates microglia metabolic dysregulation and dysfunction (68). Lactate-derived lactylation modification on core histones has been demonstrated as a novel histone marker by several investigators. Lactylation modifications of histones preferentially affect enzymes involved in essential metabolic pathways such as carbohydrate, amino acid, lipid and nucleotide metabolism. Lactate promotes cell proliferation and migration in part through histone lactylation, particularly H3K18la, and this process is mediated by TTK protein kinase (TTK) and serine/threonine kinase B (BUB1B), which in turn enhances glycolysis and increases lactylation (7). This study establishes a positive feedback loop between glycolysis, histone lactylation, and cell cycle genes, which adds a novel mechanism for PDAC and provides clues for the treatment of PDAC. However, strategies involving histone lactylation therapy to overcome and alter the molecular landscape of PDAC have not yet been realized. Acetyl coenzyme A, a donor for histone acetylation, is dependent on intracellular acetyl coenzyme A availability, which is dynamically regulated by glucose availability in the TME (69). ATP Citrate Lyase(ACLY) is a metabolic enzyme found in cytoplasmic lysates and the nucleus that cleaves citrate salt to produce acetyl coenzyme A and plays an important role in the regulation of histone acetylation. In PDAC, high levels of histone acetylation are associated with low survival. High glucose conditions induce BMI1 expression via ATP-citrate lyase-dependent acetyl coenzyme A. Bmi1 regulates the Warburg effect in PDAC cells and enhances immunosuppression of TME by targeting HK2 (70). Frequent histone lysine succinylation is a newly identified histone modification that can be regulated by KAT2A histone succinyltransferase. KAT2A is highly expressed in human PDAC specimens and positively correlates with survival of patients with PDAC in both late and short-term stages. KAT2A regulates H3K79 succinylation in the promoter region of YWHAZ (encoding 14-3-3ζ) to promote YWHAZ mRNA and 14-3-3ζ expression, which prevents β-linker degradation and thus promotes tumor proliferation (71). The interplay between histone modification and metabolism reflects the cell’s delicate balance between energy perception and regulation of gene expression. In disease, disruption of this balance leads to the persistence of a “metabolic memory” that drives malignant phenotypes. In the future, multi-omics technologies (e.g., combined metabolome-epigenome analysis) will be required to analyze the temporal and spatial dynamics and develop microenvironment-specific targeted therapies to break the metabolic-epigenetic vicious cycle of tumors.

In PDAC, autocrine insulin-like growth factor-1 (IGF-1) and paracrine insulin stimulate the IGF-1 receptor (IGF1R) and insulin receptor (IR) to increase tumor growth and glycolysis. Cavity protein-1 (cav-1), a protein that coexists with insulin-like growth factor receptor (IGF1R) and insulin resistance (IR), stimulates IGF1R/IR and glycolysis in cancer cells and triggers a malignant state in tumor carriers (72). Integrin subunit alpha 3 (ITGA3) is a cell surface adhesion protein involved in tumor progression. ITGA3 enhances glycolysis to promote pancreatic cancer growth and metastasis by increasing HIF-1α and c-Myc expression in a Col1-dependent autocrine manner (73).

### 2.4 Mitochondrial dysfunction

Mitochondria play a central role in energy production and respiration (oxidative phosphorylation), and the contribution of this biology to survival increases under metabolic stress, including low glucose. Pancreatic cancer cells rely on efficient ATP production under adverse conditions to drive pro-survival pathways. For adeno-ductal metaplasia (ADM), the first step in pancreatic carcinogenesis, it has been revealed that the switch from oxidative phosphorylation to glycolysis attenuates ADM formation by blocking the metabolic switch in ADM. In addition, ab initio synthesis of serine and glutathione requires mitochondrial metabolism, whereas ATP production does not. C-Myc mediates an increase in GSH intermediates in ADM, whereas inhibition of GSH synthesis suppresses ADM development (74). Mitochondrial programs are down-regulated in the partially immune population of pancreatic cancer, relative to normal mitochondria in a healthy pancreas. Although granulocytes, B cells, and CD8+ T lymphocytes all downregulated oxidative phosphorylation, the mechanism by which this occurred was cell-type specific, and the expression pattern of the electron transport chain complex was sufficient to recognize immune cell types without the use of lineage markers (75).Silencing of cytidine deaminase (CDA) in PDAC cells altered the levels of several metabolites produced by mitochondria, but also reduces mitochondrial respiration and ATP production. It reconnects cancer cell metabolism to glycolysis.CDA promotes mitochondrial biogenesis independently of its known catalytic activity.CDA has new and unexpected effects on mitochondrial biology and OXPHOS in PDAC cells, which establishes a link between enzymes involved in pyrimidine production and mitochondrial function (76). The pyruvate dehydrogenase complex (PDC) plays a Carbohydrate metabolism plays a central role linking cytoplasmic glycolysis to the TCA cycle. PDC is a conserved E1-E2-E3 dehydrogenase, with PDHA1 and PDHB heterotetramers functioning as E1 subunits. Hypoxia induces the expression of PDHK1 and PDHK3 and hyperphosphorylates PDHA1. PDC plays a role in the metabolic reprogramming of pancreatic cancer by integrating oncogenic and environmental signals, thereby supporting tumor growth (77). Overexpression of wild-type isocitrate dehydrogenase 1 (IDH1) in PDAC cells promotes PDAC cell survival by supporting mitochondrial function and antioxidant defense (74).

**Figure 2:**
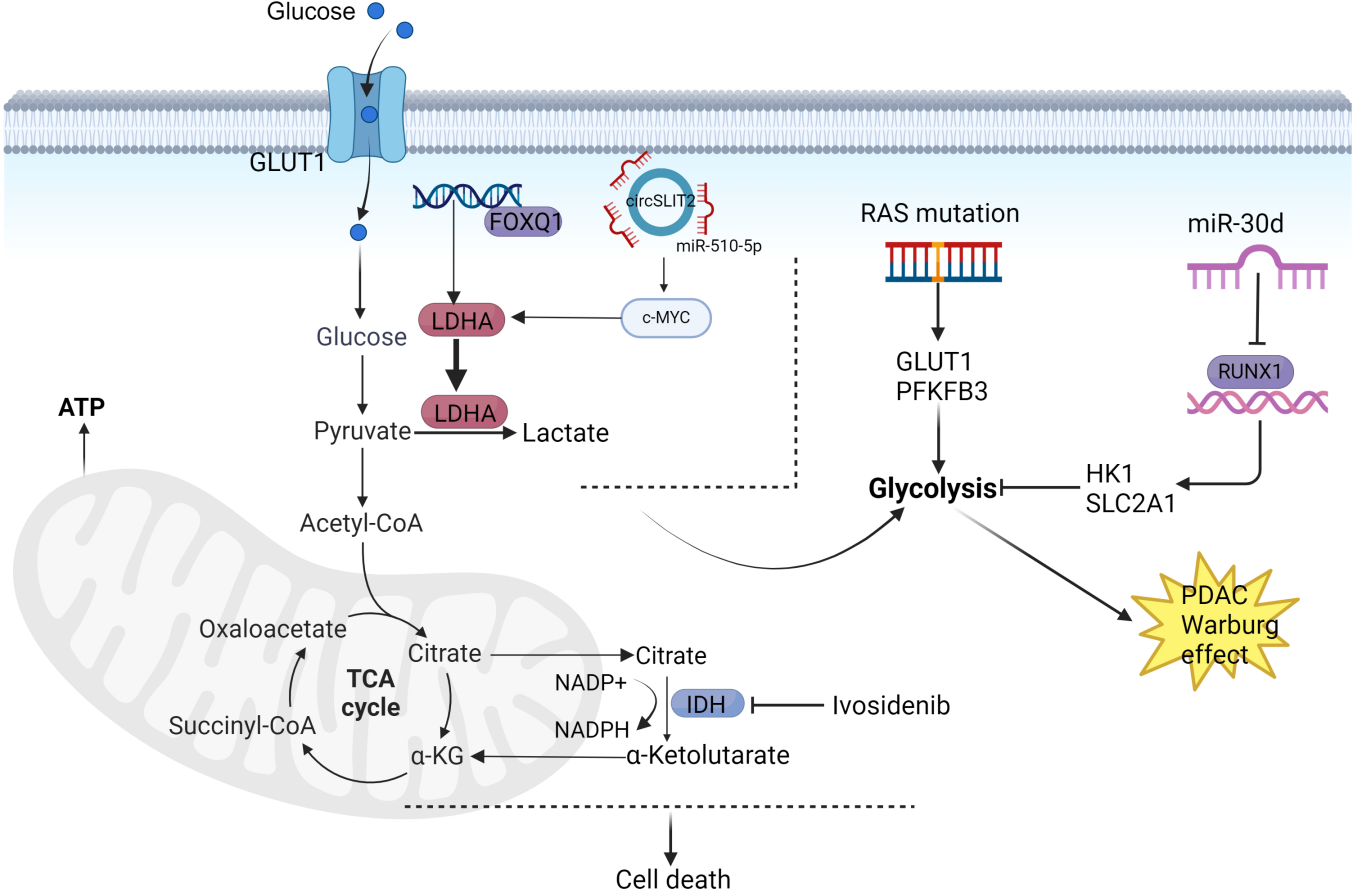
Mechanisms of PDAC glucose metabolism reprogramming. PFKFB3 and GLUT2 mediate KRAS oncogenic signaling with the Warburg effect, thereby promoting PDAC progression. MiR-30 functions as a tumor suppressor in PDAC by inhibiting pancreatic tumorigenesis through suppression of aerobic glycolysis. CircSLIT2 participates in PDAC carcinogenesis via the circSLIT2/miR-510-5p/c-Myc/LDHA axis, which regulates aerobic glycolysis and malignant transformation. FOXQ1 enhances LDHA transcriptional activation to facilitate aerobic glycolysis in PDAC. PDAC cells overexpressing wild-type isocitrate dehydrogenase 1 (IDH1) demonstrate improved survival through mitochondrial functional maintenance and reinforcement of antioxidant defense systems. Created in https://BioRender.com.

Mutations in mitochondrial DNA (mtDNA), particularly mtDNA encoding OXPHOS-related proteins, result in altered mitochondrial function, including OXPHOS (78). An early study sequencing the complete mtDNA genome of PDAC cells reported a significant increase in intracellular mass in mtDNA mutant cells compared to normal cells, suggesting more mtDNA somatic mutations (79). During the cellular life cycle, mitochondrial morphology is continuously remodeled through fission and fusion events to adapt to the cellular environment (80, 81). Dynamin-related protein 1 (DRP1), mitochondrial fission protein 1 (FIS1), and mitochondrial fission factor (MFF) are the most relevant proteins involved in mitochondrial fission in mammalian cells (82, 83). Mitochondrial fusion is mainly controlled by mitochondrial fusion protein 1 (MFN1), mitochondrial fusion protein 2 (MFN2), and OPA1 (optic nerve atrophy 1) control (84). In recent years, there has been increasing evidence of a strong link between mitochondrial fission and fusion dysfunction and cancer (85). Dysregulated expression of DRP1, DRP1, and MFN2 has been observed in several types of cancers, including hepatocellular carcinoma (86), breast carcinoma (87), and lung carcinoma (88), DRP1 is significantly up-regulated in pancreatic cancer cells and tissues, whereas knockdown of DRP1 strongly induced apoptosis in PDAC cells. Knockdown of DRP1 significantly reduced glucose consumption and lactate production in PDAC cells, and after replacing glucose with galactose in the culture medium, galactose was found to significantly abrogate the tumor-promoting effect of DRP1 on the growth and invasive potential of SW1990 and AsPC-1 cells (89). The glycolytic reprogramming metabolic map of PDAC is illustrated in **Figure 2**.

### 2.5 Interactions in the tumor microenvironment

The interaction of stromal cells, immune cells and malignant cells creates a tumor microenvironment (TME) that can exert physical stress, oxidative stress, nutrient deprivation, competition, hypoxia and immune surveillance on cancer cells (90). Notably, the TME of PDAC has a strong connective tissue proliferative response and low vascular density, which reduces nutrient and oxygen delivery capacity and leads to enhanced glycolysis and lactate deposition (91). In the TME, fibroblasts and immune cells actively support the cancer cells to ensure that tumor cell development is sustained even in the absence of adequate blood vessel formation.

#### 2.5.1 Hypoxia

Several studies in recent years have identified hypoxia as a major inducer of glycolytic conversion in tumors (92). Ubiquitin-specific protease 25 (USP25) is a major regulator of glycolysis by regulating hypoxia-inducible factor-1α (HIF-1α) stability and transcriptional activity (93). Increased expression of key genes of glycolysis and HIF-1α was verified in Neuromedin U (NMU) stimulation, which resulted in the activity of key enzymes PK and LDH increased and promoted the production of lactate by tumor cells, which could induce the differentiation of tumor-associated macrophages to an M2-like phenotype through the activation of HIF-1α, and induced the expression of arginase 1 and vascular endothelial growth factor to promote tumor invasion, metastasis, and angiogenesis (94). Hypoxia-induced circRNF13, mediated by HIF-1α and EIF4A3, promoted PDAC tumor progression and glycolysis, circRNF13 has the potential to be a prognostic biomarker and therapeutic target for PDAC (95). Hypoxia-induced exosome circPDK1 promotes pancreatic cancer glycolysis through c-Myc activation by regulating the miR-628-3p/BPTF axis and degradation of BIN1 (96). Alkaline leucine zipper and inclusion of the W2 structural domain protein 1 (BZW1) promote PDAC metabolic stress resistance and glycolysis by controlling eIF2α phosphorylation-mediated translation of HIF-1α and c-Myc (97). Lysyl oxidase-like 2 (LOXL2) is a hypoxia-responsive gene, and there exists a positive feedback loop between LOXL2 and HIF-1α that promotes glycolytic metabolism under hypoxic conditions. In addition, LOXL2 combines with the Warburg effect in PDAC to promote tumor growth and metastasis (98). Hypoxia and endoplasmic reticulum stress lead to overexpression of endoplasmic reticulum oxidoreductase 1α (ERO1L) in PDAC. Researchers confirmed the modulation of the Warburg effect by ERO1L using bioinformatics analysis and functional analysis, and inhibition of tumor glycolysis partially eliminated the growth-promoting activity of ERO1L (99).

#### 2.5.2 Cancer-associated fibroblasts

Oxygen depletion accompanying tumor proliferation poses a barrier to the nutritional requirements of tumors, and the TME undergoes adaptive changes to meet the increased biosynthetic demands of tumor proliferation through multiple metabolic pathways. In response to these complex microenvironmental changes, metabolic reprogramming in CAFs induces a shift in energy production from mitochondria to glycolytic sources, which contributes to the formation of a hypoxic and acidic TME that supports tumor growth in multiple dimensions (10). Significant expression of MCT1, fumarate hydratase and succinate dehydrogenase in pancreatic cancer cells suggests a metabolic coupling between CAFs and tumor cells. Pancreatic CAFs can alter metabolism and communicate and respond to cancer cell migration and invasion. This may be an important mechanism to promote tumor progression in a non-vascular manner in the tumor microenvironment. The mechanisms by which CAFs remodel metabolic shifts require further analysis (100). In addition, CAFs from platinum-resistant patients were found to significantly enhance oxaliplatin resistance in pancreatic cancer cells through the secretion of IL-8 and extracellular vesicles, thereby promoting non-homologous end-joining (NHEJ)-dependent DNA repair mechanisms (101). Hu et al. revealed that CAFs induce gemcitabine resistance in pancreatic cancer through aerobic glycolysis mediated by monocarboxylic acid transporter proteins, underscoring the potential of targeting the metabolic connectivity of CAFs in cancer cells to enhance chemotherapeutic outcomes (102). Nevertheless, the impact of glycolytic regulation of CAFs on pancreatic cancer drug resistance remains underexplored, highlighting the need for in-depth studies.

#### 2.5.3 Immune microenvironment

A typical feature of PDAC is the immune-tumor microenvironment, where increased glycolysis in PDAC tumors leads to a low-glycemic, high-lactate TME, which suppresses immune cell function.

Stimulated by pathological factors, bone marrow-derived pluripotent stem cells differentiate into different types of T lymphocytes, which perform the appropriate functions. T lymphocyte activation is thought to drive the initial increase in glucose uptake, which is accompanied by an upregulation of aerobic glycolysis (103). However, when glycolysis rates are low, chronic nutrient deficiencies may lead to T lymphocyte energy. One study found that Dickkopf-related protein 3(DKK3) increased glucose levels, promoted CD4+ T lymphocyte proliferation and decreased apoptosis levels (104). Pathways associated with oxidative metabolism promote effector functions in immune cells (105). A previous study also reported that lactate inhibits cytokine production triggered by T lymphocyte receptors and impairs lysate granule cytotoxicity in cytotoxic T lymphocytes (106). Neuromedin U (NMU) impairs the biological function of CD8+ T lymphocytes in the PDAC tumor microenvironment in an NMUR1-dependent manner, whereas blockade of lactate production by tumor cells restores NMU-mediated inhibition of the antitumor activity of CD8+ T lymphocytes, which provides a new idea for immunotherapy (94).

Tumor-associated macrophages (TAMs) are key elements of immune infiltration within tumor tissues. A large proportion of TAMs are recruited and polarized from the peripheral blood via cytokines and chemokines secreted by tumor and stromal cells. Macrophages can be classified into two types: classically activated M1 type and selectively activated M2 type, and in TMEs, the majority of TAMs differentiate into the M2 phenotype, which promotes tumor progression, including tumor growth, metastasis, and angiogenesis (107). Pyruvate metabolic remodeling and glycolysis in PDAC are closely related to the M2 type (108). In one study, it was determined that more TAMs were infiltrated in tumor samples of pancreatic cancer with intense glycolysis than in tumor samples of pancreatic cancer with attenuated glycolysis by analyzing the immunohistochemical results of the patients’ preoperative PET-CT images and postoperative tumor sample sections. The data also suggest that TAMs enhance tumor glycolysis to promote malignant progression of pancreatic cancer. TAMs promote tumor progression primarily through the secretion of a variety of cytokines, chemokines, and other elements that impact both the tumor and TME (109). For example, TAMs secrete IL-8 to promote GLUT3 expression in PDAC cells and enhance tumor glycolysis *in vitro* and *in vivo (* 109). Tumor extracellular vesicles (EVs) are involved in macrophage polarization, and LINC00511, encapsulated in pancreatic cancer (PDAC) cell-derived EVs, accelerates glycolysis and promotes mitochondrial oxidative phosphorylation in PDAC cells through macrophage polarization, thereby inducing invasion and migration of PDAC cells (110). This provides new novel ideas for developing immune and targeted metabolic therapies for PDAC.

Dendritic cells (DCs) use glucose to support their effector functions, while conventional dendritic cells (cDCs) consist of the cDC1 and cDC2 subpopulations. These two subpopulations play a key role in anticancer immunity by promoting the activation of cytotoxic CD8+ T lymphocytes and CD4+ T lymphocytes, respectively. Relative to other tumor types, PDAC has a lower number of dendritic cells in the TME and poorer antigen-presenting capacity. Increased glycolysis in PDAC tumors results in a low-glucose, high-lactic acid tumor microenvironment (TME), which suppresses immune cell function, particularly dendritic cells (DCs) and CD8+ T lymphocytes (111). However, this study was conducted in mice, and future studies should evaluate the effects on human-derived DCs to better elucidate the detailed mechanisms underlying the attenuated DC antigen-presenting function in low-glucose and high-lactate environments. Currently, studies on DC antigen-presenting function have focused on immune cell interactions and the effects of cytokines (112). The effect of metabolic reprogramming in tumors on this antigen-presenting function is unknown.

Myeloid-derived suppressor cells (MDSCs) are a heterogeneous population of cells that arise in a range of pathological conditions, such as cancer, inflammation, and infection, and show a remarkable ability to suppress T lymphocyte responses (113). During maturation and activation, these tumor-derived MDSCs exhibit increased central carbon metabolism, including glycolysis, PPP, and TCA cycling. Tumor-derived MDSCs exhibit a high degree of glycolysis upregulation, and their metabolite phosphoenolpyruvate protects MDSCs from apoptosis and contributes to their survival. Inhibition of glycolysis by 2-deoxyglucose was shown to inhibit MDSCs differentiation from precursor cells, whereas enhancement of glycolysis with metformin significantly rescued rapamycin-induced MDSC decline (114). In the tumor microenvironment, glycolytic activation induces the expansion of bone marrow-derived myeloid precursor cells into MDSCs, which may be inhibited by the mTOR inhibitor rapamycin. Accumulating evidence now suggests that immune cells have a high degree of metabolic plasticity, which can alter their differentiation and function according to the desired environment.

Tumor-associated neutrophils (TANs) are neutrophils that infiltrate into the TME, including areas of tumor infiltration, tumor periphery, or areas close to blood vessels and metastatic sites (115). They can promote or inhibit tumor progression (116). These functions include tumor cell killing, promotion of inflammation, tissue remodeling, regulation of angiogenesis, and modulation of the immune response (117, 118). Neutrophil function in TME is also affected by HIF-1α, which helps neutrophils adapt to hypoxic stress and promotes anaerobic glycolysis (119). In addition, glycolysis induced by LDHA and basic helix-loop-helix family member e40 (BHLHE40) contributes to the pro-tumorigenic function of the TANs subpopulation (120). These findings suggest that targeting metabolic pathways, including glycolysis, in TANs within TME may offer a potential therapeutic strategy to modulate their function and enhance anti-tumor immunity.

#### 2.5.4 Extracellular matrix

**Figure 3:**
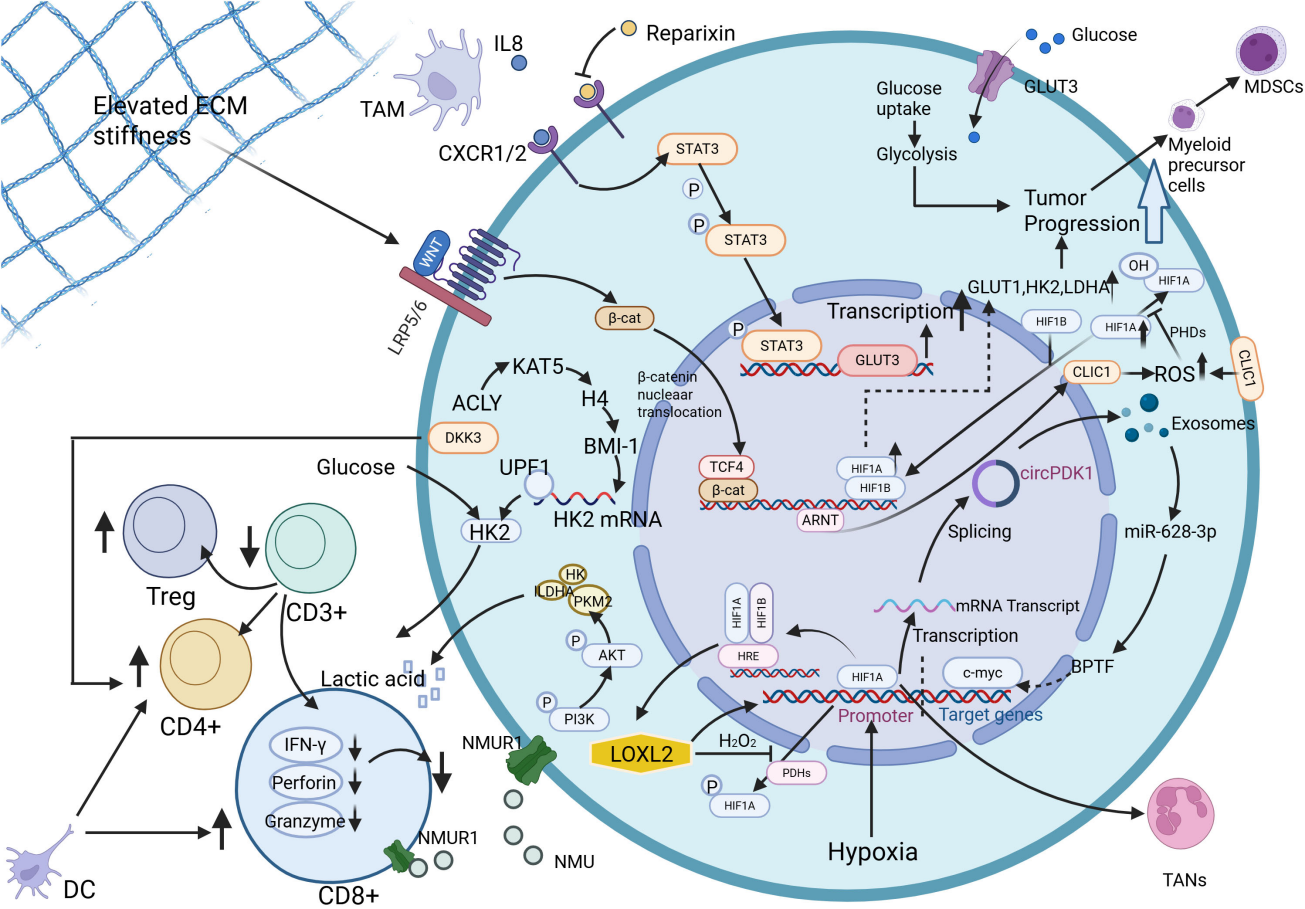
TME orchestrates glycolytic reprogramming in PDAC through interconnected molecular cascades. Neuromedin U (NMU) activates pyruvate kinase M2 (PKM2) and lactate dehydrogenase A (LDHA), driving excessive lactate production that stabilizes HIF-1α to polarize tumor-associated macrophages (TAMs) toward immunosuppressive M2 phenotypes. Concurrently, NMU suppresses CD8+ T-lymphocyte antitumor responses via NMU receptor 1 (NMUR1), while pharmacological blockade of lactate synthesis reverses this immunosuppressive effect. Hypoxia-inducible exosomal circPDK1 fuels glycolytic flux by dual mechanisms: sponging miR-628-3p to upregulate bromodomain PHD finger transcription factor (BPTF) and destabilizing bridging integrator 1 (BIN1), thereby amplifying c-Myc-driven metabolic reprogramming. A self-reinforcing circuit between lysyl oxidase homolog 2 (LOXL2) and HIF1α perpetuates hypoxia-adapted glycolysis through coordinated transcriptional activation of glycolytic enzymes. TAM-derived interleukin-8 (IL-8) upregulates glucose transporter 3 (GLUT3) expression, enhancing glycolytic output and tumor progression both *in vitro* and *in vivo*. Dickkopf-related protein 3(DKK3) increases glucose levels, promotes proliferation of CD4+ T lymphocytes, and decreases apoptosis levels. Dendritic cell(DC) promotes the activation of cytotoxic CD8+ T lymphocytes and CD4+ T lymphocytes, which play a key role in anti-cancer immunity. Activation of glycolysis induces the expansion of bone marrow-derived myeloid precursor cells into Myeloid-derived suppressor cells(MDSCs). Tumor-associated neutrophils(TANs) function in TME is affected by HIF-1α. Furthermore, mechanotransduction of extracellular matrix (ECM) stiffness activates the Wnt/β-catenin/TCF4 axis, elevating chloride intracellular channel 1 (CLIC1) expression to sustain glycolysis-dependent proliferation. Created in https://BioRender.com.

Interestingly, untransformed extracellular matrix (ECM) stiffness is involved in the complex regulation of metabolic reprogramming, including carbohydrate metabolism, with chloride intracellular channel 1 (CLIC1) acting as a bridge between tumor stromal stiffness and the Warburg effect in PDAC. Pancreatic cancer cells were found to sense ECM stiffness and activate the Wnt/b-catenin/TCF4 signaling pathway, leading to upregulation of CLIC1 expression, which promotes glycolysis-dependent tumor growth (121). TME drives glycolytic reprogramming in PDAC, as schematically illustrated in **Figure 3**.

### 2.6 Pancreatic cancer treatment resistance

Despite recent advances in early diagnosis and therapeutic strategies, the outcome of pancreatic cancer remains unsatisfactory. The mechanisms of therapeutic resistance in pancreatic cancer are complex and diverse, involving the tumor microenvironment, metabolic reprogramming, genetic mutations and other factors. Therapeutic resistance not only leads to clinical treatment failure, but also greatly affects the survival prognosis of patients.

Resistance to gemcitabine (GEM) in PDAC is an important factor leading to ineffective chemotherapy, delayed treatment and poor prognosis. Glycolysis is thought to be one of the key mechanisms of gemcitabine resistance, which competitively inhibits gemcitabine activity by promoting the accumulation of deoxycytidine triphosphate (dCTP) in PDAC. Although postoperative chemotherapy is the standard treatment option for patients with pancreatic cancer, no study to date has demonstrated that it significantly improves the long-term prognosis of patients. With increasing resistance to chemotherapy in pancreatic cancer, it is difficult for a single chemotherapy regimen to alter a patient’s prognosis. Tumor glycolytic pathways have been shown to play an important role in promoting chemotherapy resistance. Therefore, chemotherapeutic strategies combining metabolic modulation have gradually attracted the attention of researchers. However, therapeutic tools targeting tumor metabolism alone are still insufficient because metabolic changes alone are not sufficient to alter the composition of the TME, and the active components in the TME still support tumor progression. Therefore, targeting synergistic changes in tumor cell metabolism and TME to enhance the efficacy of chemotherapy has become an important direction in current therapeutic strategies.

In response to gemcitabine therapeutic resistance, researchers have proposed a number of strategies that could improve sensitivity. Targeting the glycolytic pathway can increase the sensitivity of SETD2-deficient PDAC to gemcitabine. SETD2-deficient PDAC is highly dependent on glycolysis, particularly through upregulation of GLUT1 to promote tumor growth. Therefore, the combination of GLUT1 inhibitors with gemcitabine may provide a new therapeutic strategy for patients with SETD2-deficient PDAC (27). ROCK1, a member of the Rho-associated coiled-coil protein kinases (ROCKs), plays a key role in pancreatic cancer metastasis and progression. ROCK1 promotes, through the c-Myc/PFKFB3 signaling pathway, the glycolysis in pancreatic cancer cells and drives tumor growth. Knockdown of ROCK1 can effectively inhibit pancreatic cancer growth *in vivo* and increase tumor sensitivity to gemcitabine, providing a new strategy for clinical treatment (122). LIPH (Lipase H), a membrane-associated phosphatidic acid-selective phospholipase A1a, is able to maintain the stability of ALDOA through activation of the LPA/LPAR axis, and directs ALDOA stability, directly connects PDAC cells to the tumor microenvironment, and promotes aberrant aerobic glycolysis. Xenograft models showed that high LIPH-expressing PDAC were sensitive to gemcitabine treatment without triggering significant side effects, suggesting its potential as a combination therapy (123). Overexpression of wild-type isocitrate dehydrogenase 1 (IDH1) by PDAC cells promotes PDAC cell survival by supporting mitochondrial function and antioxidant defenses. IDH1 promotes PDAC cell survival by generating α-ketoglutarate and NADPH to neutralize ROS and resist oxidative stress after chemotherapy. Ivosidenib inhibition of wild-type IDH1 when combined with conventional chemotherapeutic agents such as FOLFIRINOX enhances its efficacy in a mouse model of PDAC, providing a promising clinical therapeutic option (74, 124). Pharmacological doses of vitamin C are able to inhibit citrate synthase (CS) activity and reduce the level of glucose-derived citrate, which in turn inhibited tumor proliferation and significantly enhanced the response to gemcitabine. In addition, reduced citrate utilization correlated with the overall inhibition of glycolysis, providing a new idea to improve patients’ chemotherapeutic response by regulating citrate metabolism (125). Furthermore, human equilibrium nucleoside transporter protein 1 (hENT1) was able to effectively reverse gemcitabine-induced resistance by inhibiting glycolysis and regulating HIF-1α in pancreatic cancer, further demonstrating that the regulation of glucose transporter’s potential role in PDAC drug resistance (126).

Chemotherapy only slightly extends their life by a few months; immunotherapy has revolutionized the treatment of pancreatic cancer. Immunotherapy is now the focus of pancreatic cancer treatment. However, this recalcitrant tumor evades immune detection primarily through the secretion of immunosuppressive factors such as transforming growth factor-β (TGF-β), the creation of an immunosuppressive environment devoid of T-lymphocytes, and the expression of immune checkpoints such as programmed death ligand 1 (PD-L1) and PD-L2. The microenvironment of pancreatic cancer is characterized by extensive nodal hyperplasia, scarcity of effector T-lymphocytes, and the presence of helper T-lymphocytes 2 as the primary tumor cell. scarcity, and an immune phenotype dominated by helper T lymphocytes 2 cells, all of which contribute to cancer cell evasion of immune surveillance (127). Current immunotherapies have had limited success in improving the survival of patients with PDAC. Immunoresistance of PDAC to immunotherapies can be attributed to its low mutational load and hostile TME characterized by fibrosis, hypoxia, and immunosuppression (128, 129).

### 2.7 Subtype-specific reprogramming of glucose metabolism

Glycometabolic reprogramming serves as a key feature of PDAC, and the manifestation and function of this metabolic alteration may differ significantly between subtypes (130). Therefore, clarifying such differences is important for precision therapy. In 2015, Yu et al. categorized PDAC into glucose- and glutamine-dependent metabolism based on the expression of metabolism-related proteins. Among the glucose-dependent metabolism, there are Warburg-type, reverse Warburg-type, mixed-type, and null-type. Warburg-type and mixed-type consist of tumors that are more metabolically active, biologically invasive, and have a poorer prognosis, while reverse Warburg-type and null-type consist of metabolically inactive, biologically noninvasive, and prognostically better tumors (131). In 2017, Nicolle et al. identified two subtypes motivated by metabolic correlations and defined them as Basal and Classical. Notably, the basal phenotype identified in this study was associated with upregulation of genes related to the glycolytic pathway; whereas the classical subtype exhibited a general increase in redox-related metabolites, this study suggests that targeting the metabolic profile of our transcriptomic subtype for treatment may also be an active and feasible approach (132). In 2022, Rodriguez et al. identified three different types of glycolysis based on specific glycolysis genes. The fucoidan glycosylation subtype is characterized by increased expression of genes involved in fucoidan glycosylation (GMDS, etc.) and O-glycosylation (GALNT4, etc.). Basal subtype is characterized by higher expression of genes encoding galactose lectin-1 and the mucins MUC4 and MUC16. Mixed/low tumor content, on the other hand, is characterized by a lower content of tumor cells (133). In 2023, Li et al. classified pancreatic cancer into four TAM2-associated metabolic subtypes based on the expression profiles of genes related to pyruvate and glycolysis metabolism by proteomic and metabolomic analysis:quiescent, pyruvate, glycolysis/gluconeogenesis and mixed (108). The KEGG pathway of the quiescent subtype is dominated by glucose, amino acid and lipid metabolism. The KEGG signaling pathway of the pyruvate subtype is closely related to the MAPK and CAMP signaling pathways. The KEGG pathway of the GG subtype is enriched in glucose metabolism and is characterized by exogenous substance metabolic processes, detoxification and homeostasis in tissues. The KEGG pathway of the mixed subtype is involved in immune-related biological processes and signaling molecules, and is characterized by extracellular matrix, antigen presentation and serine/threonine kinase signaling pathways.

**Figure 4:**
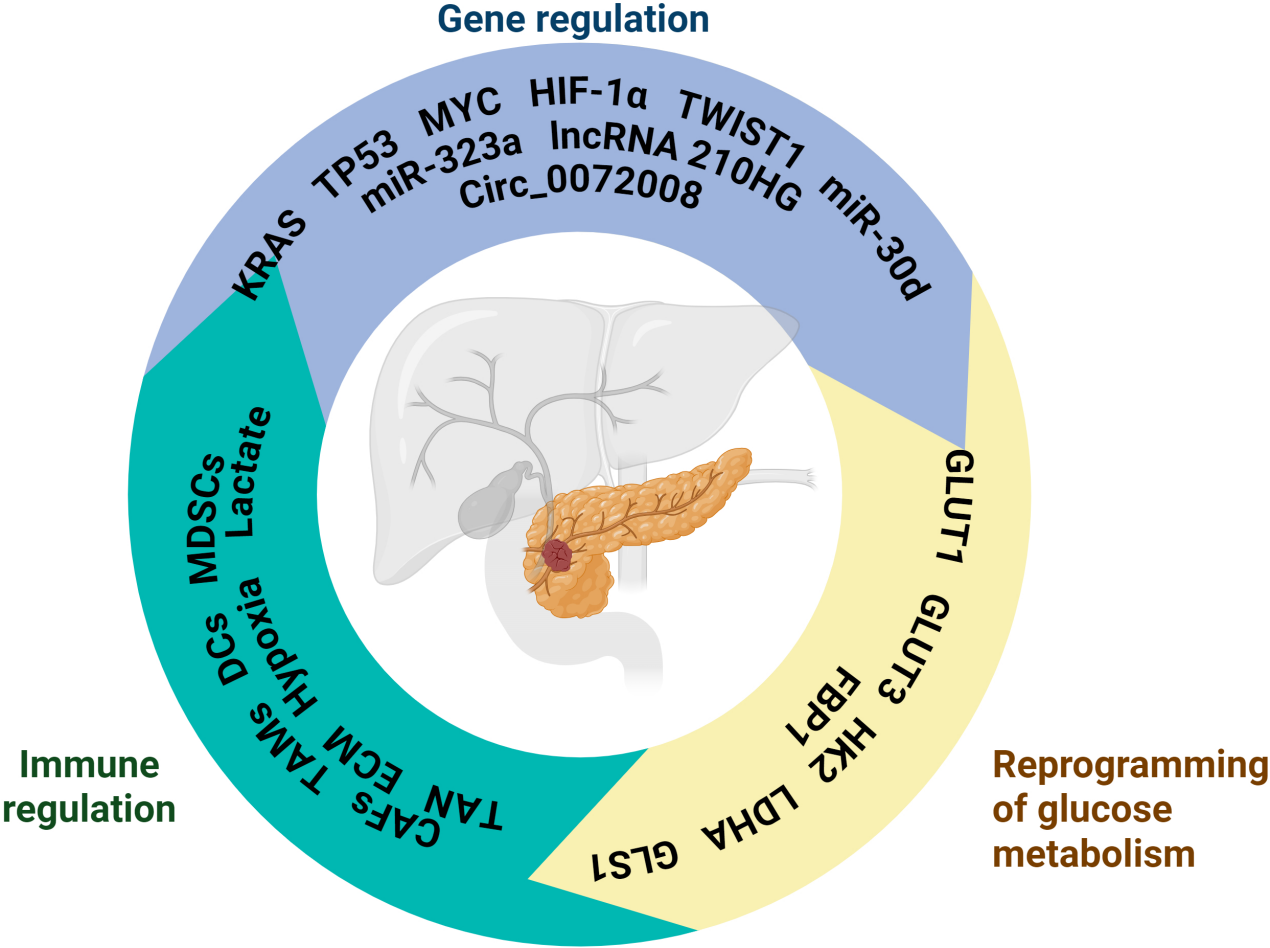
The schematic is centered around pancreatic cancer, presenting its related key regulatory mechanisms, covering three core aspects: Gene regulation involves genes such as KRAS, TP53, MYC and non-coding RNAs such as miR-323a and miR-30d, which regulate the physiological or pathological process of pancreatic cancer at the level of gene expression; Immunomodulation contains MDSCs, Lactate, TANs and other immune-related cells and molecules, which are involved in the immunoregulation of the pancreatic cancer microenvironment and affect the immune response; Glucose metabolism reprogramming involves molecules such as GLUT1 and HK2, which reflect altered glucose metabolism pathways in pancreatic cancer cells and are closely associated with metabolic abnormalities in disease states. Created in https://BioRender.com.

However, there are differences and lack of consistency in the criteria and methods for classifying PDAC subtypes in different studies, which increases the difficulty of clinical translation of metabolically targeted therapeutic strategies. Moreover, the metabolic state of PDAC is highly dependent on the regulation of the tumor microenvironment, for example, factors such as hypoxia and nutritional deficiencies can lead to changes in the metabolic state, and even a single tumor may exhibit multiple metabolic states in possibly different regions. Large discrepancies remain regarding the causality of the relationship between isoforms and metabolism, and current evidence does not yet allow a clear distinction to be made between whether transcriptional isoforms determine metabolic dependence or whether metabolic status, in turn, shapes the transcriptional profile. Therefore, a deeper understanding of PDAC subtype-specific reprogramming of glucose metabolism will not only help to reveal the nature of its tumor biology, but may also provide a stratification basis for metabolically targeted therapeutic strategies. The interplay among metabolic reprogramming, genetic regulation, and immune modulation in PDAC is schematically summarized in **Figure 4**.

### 2.8 Specificity of reprogramming of PDAC glucose metabolism

Compared to other cancers, the reprogramming of glucose metabolism in PDAC exhibits highly unique adaptive strategies, features that directly derive from its oncogenic driver mutations (e.g., Kras) and the extremely harsh tumor microenvironment. Mutations in the oncogene Kras are hallmark events in PDAC and play a key role in tumor initiation. Several studies have found that Kras G12D controls tumor metabolism by stimulating glucose uptake and directing glucose intermediate metabolites to glucosamine biosynthesis and the PPP. These studies also revealed that the oncogene Kras promotes ribose biosynthesis, and that Kras G12D drives glycolytic intermediates to the non-oxidative pentose phosphate pathway, thereby uncoupling ribose biosynthesis from NADP/NADPH-mediated redox regulation (24, 134). Glu generates reducing equivalents of NADPH driven by oncogenic Kras. Kras activates the GOT2-GOT1-ME1 pathway and initiates a ROS detoxification program dependent on nuclear factor (erythropoietin-derived 2-like 2, NFE2L2). Mutant Kras persistently activates this antioxidant program to inhibit ROS and enhance pancreatic tumorigenesis. Glutamine is not only involved in the TCA cycle to generate energy, but also supports cancer cell proliferation by regulating carbon supply, which is unique to PDAC (135). Unlike other solid tumors, pancreatic cancer has abundant stromal cells and abundant extracellular mesenchyme, but lacks angiogenesis, resulting in persistent and severe hypoxia within the tumor. The hypoxic microenvironment has a wide range of effects on the biological behavior or malignant phenotype of pancreatic cancer, including metabolic reprogramming, cancer intervention, invasion and metastasis, and pathologic angiogenesis, which collectively contribute to pancreatic cancer progression and treatment resistance. Intratumor hypoxia drives the aforementioned biological processes in pancreatic cancer through various mechanisms including, but not limited to, maintenance of redox homeostasis, activation of autophagy, epigenetic regulation, and induction of hypoxia-inducible factors (129, 136). Moreover, the researchers found that hypoxia due to low blood vessel density and vascular defects can promote the metastatic potential of cancer cells (137). Activated stromal fibroblasts, known as CAF, also play a major role in PDAC progression. The autocrine/paracrine effects of CAF on neighboring stromal and epithelial cancer cells confer tumor cell invasiveness, cancer stem cell (CSC) self-renewal, drug resistance, metastatic spread, and disease recurrence (138). CAF utilizes lactate dehydrogenase from glycolysis-enhanced cancer cells as fuel and exerts immunosuppressive activity in PDAC TME (10). It is these metabolic alterations that make PDAC one of the most metabolically unique and therapeutically challenging tumors. Future treatments will require the development of multidimensional combinatorial strategies targeting these unique metabolic networks.

## 3 Therapeutic strategies for targeting glucose metabolism in pancreatic cancer

### 3.1 Direct targeting of pancreatic cancer glucose metabolism

Researchers have identified a number of alternative targets for key enzymes in the glycolytic process. PDAC cells undergo a shift from mitochondrial to glycolytic metabolism, which promotes a number of cancer traits, including cell proliferation, invasion, and resistance to apoptosis (26). It has been shown that this increased rate of glycolysis is also important for fueling ATP-dependent plasma membrane calcium pumps (PMCAs) (139). PMCAs are also important for the production of glycolytic ATP in PDAC cells. This increased rate of glycolysis has been shown to be important for fueling the ATP-dependent PMCAs as well, since inhibition of glycolytic ATP production in PDAC cells leads to cytotoxic Ca2+ overload and cell death. This dependence of PMCAs on glycolytic ATP in PDAC represents a potential therapeutic pathway, as PKM2 is a major oncogenic ATP-generating glycolytic enzyme, and shikonin is the most potent and selective enzyme for PKM2. Shikonin is one of the most potent and selective inhibitors of PKM2, inhibiting glycolysis, ATP depletion, inhibition of PMCAs activity, and the resulting cytotoxic Ca2+ overload (140). Glyceraldehyde-3-phosphate dehydrogenase (GAPDH), a key glycolytic enzyme, plays an essential role in energy metabolism in cancer cells. Among the 3-bromo-4,5-dihydroisoxazole (BDHI) derivatives, compound 11 was identified as a potent hGAPDH inhibitor with higher potency than the well-known hGAPDH inhibitor koningic acid, which can be further used for the development of anticancer drugs (141). In one study, the authors obtained 14 PFKFB3 inhibitors by virtually screening a library of FDA-approved compounds. Subsequently, *in vitro* studies confirmed that lomitapide and cabozantinib S-malate exhibited excellent potential to inhibit PFKFB3. In an *in situ* pancreatic cancer model, co-administration of lomitapide and gemcitabine at a certain molar ratio demonstrated enhanced anti-tumor effects (142). tRF-19-Q1Q89PJZ directly sponges hexokinase 1 (HK1) mRNA and inhibits its expression, thereby inhibiting glycolysis in PC cells. tRF-19-Q1Q89PJZ may be a key target for PDAC therapy (143). Solute carrier family 45 member A4 (SLC45A4) is an H+-dependent sugar cotransporter protein. SLC45A4 blocks AMPK/ULK1 axis autophagy in TP53 mutant PDAC, which may be a promising biomarker and therapeutic target for TP53 mutant PDAC (144). Rho GTPase-activating protein 25 (ARHGAP25) acts as a tumor suppressor by inhibiting the AKT/mTOR signaling pathway, and may provide a therapeutic target for PDAC (145). Canagliflozin (CANA) is a sodium-glucose cotransporter protein 2 inhibitor, CANA effectively inhibits pancreatic cancer growth in a dose-dependent manner, and it has potent antitumor activity against pancreatic cancer *in vitro* and/or *in vivo*. In addition, reduced glucose uptake and lactate production and decreased mRNA levels of glycolysis-related genes, including glucose transporter protein-1 and lactate dehydrogenase A, were found in cellular assays (146). Pharmacological inhibition of ubiquitin specific protease 25 (USP25) *in vitro* and *in vivo* resulted in PDAC cell death and tumor regression, making USP25 a promising therapeutic target for PDAC (93). In animal experiments, researchers found that targeting ULK1/2 synergistically with the hexokinase I inhibitor, 2-deoxyglucose (2-DG), and the hexokinase II inhibitor, 3-bromopyruvate (3-BP), provided better therapeutic efficacy in PDAC (147).

Furthermore, oxidative phosphorylation is the main pathway by which pancreatic CSCs meet their energy requirements, and thus, the Achilles’ heel of this highly tumorigenic cell can be identified from oxidative phosphorylation (OXPHOS). The ligand complex Ru1 is not only an exciting new anticancer drug, but also serves as a molecular tool to analyze the role of OXPHOS in CSCs (148). The biguanides metformin and phenylbiguanide have been reported to inhibit the mitochondrial respiratory complex I (149–152). In a preclinical model of PDAC, both metformin and phenformin were able to inhibit tumor growth (153, 154). Metformin treatment amplified gemcitabine-induced delay in tumor growth through a less responsive pancreatic microenvironment (155). However, in high OXPHOS PDAC, the therapeutic effect of phenformin in combination with chemotherapy, was more effective than metformin (156). Rawand Masou et al. demonstrated synergistic effects between standard chemotherapy (gemcitabine) and benzbiguanide (targeting mitochondrial complex I) in high OXPHOS PDAC cells, regardless of whether they were long-established cell lines or more recently established progenitor cells from PDX. Targeting mitochondria with phenylbiguanide induces an energetic shift to a low OXPHOS state (157),which significantly enhances the antitumor effects of gemcitabine. Compound 23 (DX3-213B) has been found to be one of the most potent complex I inhibitors reported to date. DX3-213B disrupts adenosine triphosphate (ATP) production, inhibits complex I function, and leads to inhibition of pancreatic cancer cell growth (158). NADH dehydrogenase (ubiquinone) 1 alpha subcomplex 4 (NDUFA4) affects the mitochondrial respiratory pathway, and high levels of NDUFA4 correlate with low survival. Knockdown of the NDUFA4 gene decreased the rate of oxygen consumption, cytosolic adenosine triphosphate levels, mitochondrial complex IV activity, and the protein levels of COX6C and COX5B, significantly inhibited the growth of SW1990 cell-derived xenograft tumors *in vivo (* 159). Amer Alasadi et al. synthesized a new mitochondrial uncoupler, MB1-47, which resulted in (1) accelerated pyruvate oxidation and TCA conversion (2); increased AMP/ATP and ADP/AMP ratios; and (3) decreased rates of nucleotide and glycanucleotide synthesis. In addition, MB1–47 inhibited the cell cycle at the g0-g1 phase, reduced clone formation, and inhibited the growth of mouse and human pancreatic cancer cells. *In vivo* studies demonstrated that MB1–47 inhibited tumor growth in a mouse tumor transplantation model and inhibited liver metastasis when tumor cells were transplanted intrasplenically (160). Cells overexpressing CDA were more sensitive to mitochondria-targeted drugs, and increased OXPHOS activity in cells expressing high levels of CDA revealed novel mitochondria-targeted drugs (e.g. phenelzine) in primary PDAC cultures therapeutic vulnerability (153). Unfortunately, phenelzine, which has shown antitumor activity in patient-derived xenografts, has so far been disappointing in clinical trials, including in PDAC, due to a general lack of efficacy and safety issues. ONC212 is a fluorinated imidacridone with preclinical efficacy in pancreatic and other malignancies. ONC212 impairs oxidative phosphorylation (OXPHOS) and decreases mitochondria-derived ATP production. Glucose restriction or combination with the glycolysis inhibitor 2-deoxy-D-glucose ONC212 acts synergistically and promotes apoptosis *in vitro* and *in vivo (* 161). Betulinic acid (BA) is a plant-derived natural compound with promising applications in anticancer (162). BA significantly inhibits PDAC cell viability and migration at lower doses capacity without affecting normal pancreatic cells. BA induced down-regulation of a cluster of proteins involved in mitochondrial complex 1 activity and oxidative phosphorylation, which was associated with reduced expression of RNA polymerase mitochondria (POLRMT) and cytochrome c oxidase translational activator (TACO1), suggesting that effects on mitochondrial function explain the effects of BA on PDAC cell growth and migration (163).

### 3.2 Indirect targeting of glucose metabolism in pancreatic cancer

Low extracellular pH is usually a sign of solid tumors. Tumor cells produce large amounts of lactate through glycolysis and glutamine metabolism, leading to its accumulation in the TME, which results in altered metabolism of fibroblasts, immune cells, and endothelial cells in the TME, increased acidity in the TME, and promotion of immunosuppression. Inhibition of glycolysis increases glucose availability and decreases lactate levels, which enhances DC and CD8 + T-lymphocyte function and improves antitumor responses. These findings highlight the potential of targeting tumor metabolism to enhance the immunotherapeutic effects of PDAC (111).Homologous heterotrimeric cassette C4 (HOXC4) is a member of the homologous heterotrimeric cassette family, which acts as a transcription factor in the regulation of morphogenesis. HOXC4 promotes the proliferation of PDAC cells by increasing LDHA-mediated glycolysis and increasing lactate levels in the microenvironment. Therefore, HOXC4 could be a target for PDAC therapy. From a metabolic point of view, combination therapy with TME may improve chemoresistance in PC cells (164). Berberine acts as a functional inhibitor of LDHA and inhibits LDHA and L-lactate, thereby inhibiting the progression of PDAC (7). In addition to this, berberine inhibits PanIN via the AMPK- hypoxia-inducible factor 1α pathway (165). N-hydroxyindole (NHI)-based LDH inhibitors are promising compounds because they not only inhibit cell proliferation (especially under hypoxic conditions), but also increase the sensitivity of PDAC cells to conventional chemotherapeutic agents (e.g., gemcitabine) in a synergistic manner (166, 167). Inhibition of LDH-A activity not only reduced cell proliferation, but also decreased migration and invasion of pancreatic cancer cells (168). Interestingly, inhibition of LDHA does not affect normal cells. In fact, patients with rare inborn metabolic defects and a complete lack of the LDHA gene develop myoglobinuria only after strenuous exercise (169). Thus, LDHA appears to be an ideal target for novel therapies in tumors that are highly dependent on aerobic glycolysis. LDH expression is also regulated by acetylation, and Zhao et al. demonstrated that acetylation of LDHA reduced LDH levels (170). As acetylation is reduced in PDAC tissues, this emphasizes the role of LDH as a potential target for inhibiting tumorigenesis. Furthermore, LDHA is regulated by HIF-1α, c-Myc and HER-2/neu, highlighting its key role in hypoxic adaptation and suggesting other potential targets.

**Figure 5:**
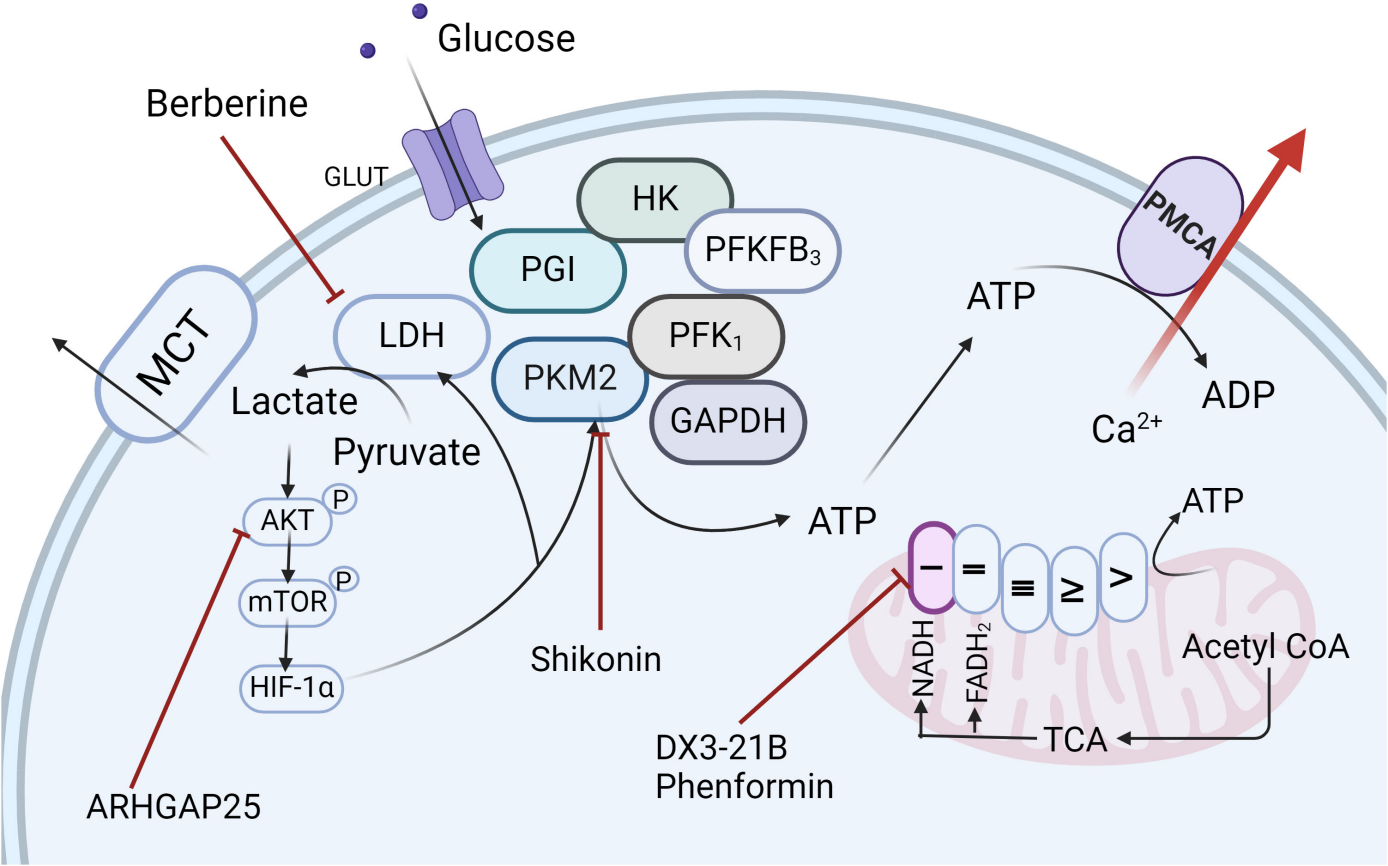
Targeted Therapeutic Strategies for PDAC. Recent research has led to the development of a range of therapeutic agents, including ARHGAP25, Berberine, Shikonin, DX3-21B, and Phenformin, which specifically target key enzymes and pathways within the glycolytic pathway, as well as mitochondrial complexes, offering promising avenues for pancreatic ductal adenocarcinoma treatment. Created in https://BioRender.com.

Targeting the tumor microenvironment of PDAC, the investigators found that hyperglycemia upregulates the Bmi1-UPF1-HK2 signaling pathway, which promotes aerobic glycolysis and lactate production in PC cells and leads to immunosuppression, and that Bmi1 is a new potential therapeutic target for patients with combined diabetes mellitus in PDAC (70). High glucose is also able to inhibit AMP-activated protein kinase signaling, leading to high expression of Bmi1, which promotes immune escape in pancreatic cancer cells. Constitutive activation of the AMPK-Bmi1-GATA2 axis can mediate MICA/B inhibition, which may serve as a therapeutic target for further intervention of immune escape in pancreatic cancer (171). Under low-glucose conditions, metformin significantly inhibited proliferation and viability and induced apoptosis in PANC-1 cells. Metformin upregulated the expression of miR-210-5p in low glucose but not in high glucose. MiR-210-5p mimics inhibited the viability of PANC-1 cells, which further enhanced the inhibitory effect of metformin, downregulated the expression of the predicted target gene of miR-210-5p, PFKFB2, and reduced the activities of PFK1 and LDH, thereby inhibit the progression of PDAC (172). Therapeutic targeting strategies for PDAC are summarized in **Figure 5**.

## 4 Immunotherapy

In recent years, researchers have proposed several innovative immunotherapeutic strategies to address the interaction mechanism between glucose metabolism reprogramming and TME in pancreatic cancer. Pancreatic cancer cells take up a large amount of glucose and secrete lactic acid through the Warburg effect, leading to glucose deprivation and increased acidity in the TME, which significantly inhibits anti-tumor immune activities such as effector T lymphocytes and NK cells. To reverse this metabolic-immune imbalance, recent research has focused on synergistic interventions of metabolic modulation and immunotherapy. Inhibition of the MCT1/4 or neutralization of lactate improves TME acidity, and preclinical studies have demonstrated that the MCT inhibitor AZD3965 in combination with a PD-L1 antibody significantly enhances T lymphocyte infiltration and inhibits tumor growth (173). In addition, glucose utilization has been found to be rate limiting for effector CAR-T lymphocyte function, and it has been demonstrated that enhancing glucose utilization by GLUT1OE enhances anti-tumor immune function (174). Macrophage recruitment was previously observed in Kras-mutated PDAC cells by chemokine secretion (175). In addition, CCL18 secreted by M2 macrophages induces aerobic glycolysis in PDAC cells and promotes tumor survival, and in turn, the increase in lactate promotes the conversion of M0 macrophages to M2 macrophages (176). Furthermore, cytotoxic NK cell activity was inhibited by lactate secreted by PDAC cells in an LDHA-dependent manner (177). Although clinical translation still faces challenges such as matrix barrier and metabolic toxicity, precision combination therapies based on metabolic-immune interaction networks (e.g., PD-1 inhibitors + metabolic modulators + matrix-targeted drugs) have become a cutting-edge hotspot for breaking through therapeutic bottlenecks. In the future, the combination of single-cell multi-omics and spatial metabolic imaging is expected to further reveal the metabolic heterogeneity of pancreatic cancer and drive the optimization of individualized immunotherapy regimens. Current drugs and clinical trials related to PDAC glycolytic reprogramming are summarized in **Table 1**.

**Table 1:** Summary of therapeutic agents and clinical trials targeting reprogramming of glucose metabolism in PDAC.

Pathways	Targets	Compounds	Combination therapies	Clinical trials	Curative status	References(source)
Glycolysis	MCT1	AZD3965		NCT01791595	Phase 1 (Completed)	(178)
	PDK	Dichloroacetate		NCT00566410	Phase 1 (Completed)	(179)
		2-deoxy-D-glucose (2-DG)	Docetaxel	NCT00096707	Phase 1 (Completed)	(180)
OXPHOS	Mitochondrial complex I	Metformin	Sirolimus	NCT02145559	Phase 1 (Completed)	(181)
		Metformin		NCT01210911	Phase 2(Completed)	(182)
		IACS-010759		NCT03291938	Phase 1 (Completed)	(183)
	TCA	CPI-613		NCT03699319	Phase 1 (Completed)	ClinicalTrials.gov
				NCT05325281	Phase 1 (recruiting)	ClinicalTrials.gov

## 5 Summary and discussion

In this study, we explored the research progress on glycometabolic reprogramming in pancreatic cancer. As a highly malignant tumor with poor prognosis, metabolic reprogramming—particularly aberrant glucose metabolism—has been demonstrated to play a pivotal role in tumorigenesis and progression. Pancreatic cancer cells remodel glucose metabolism through multiple pathways, including enhanced glycolysis, lactate production, and aerobic respiration, thereby promoting their proliferation and survival. Glycometabolic reprogramming not only provides sustained energy supply for tumor cells but also critically mediates metastasis, immune evasion, and chemoresistance. Furthermore, this metabolic adaptation is not solely intrinsic to cancer cells but involves dynamic interactions with immune cells and vascular endothelial cells within the tumor microenvironment.

With advancements in molecular biotechnology and the emergence of multi-omics data, substantial opportunities remain for elucidating the mechanisms underlying pancreatic cancer metabolic reprogramming. Key priorities include exploring the relationship between dysregulated glucose metabolism and epigenetic modifications, deciphering genetic drivers of metabolic abnormalities, linking post-reprogramming metabolite alterations to cellular phenotypic changes, and characterizing metabolite-mediated crosstalk between cancer cells and immune components. In-depth exploration of these mechanisms will likely uncover novel therapeutic targets and inform innovative treatment strategies.

Additionally, we observed that glycolytic shifts in pancreatic cancer are closely associated with transitions between aerobic and anaerobic respiratory modes. Further studies focusing on respiratory mode plasticity may unravel the complexity of pancreatic tumorigenesis and progression, offering renewed therapeutic promise.
